# Professional Advertising

**Published:** 1893-02

**Authors:** 


### Editorial.

---

### PROFESSIONAL ADVERTISING.

There is probably no question that has received less attention than this. In a quiet way in society discussions, and in the promulgation of the moral laws deemed essential for the professions, it has entered not only in the ethical code, but has also, in a measure, become part of the unwritten law that becomes such a disturbing element, at times, in the conscience of every professional man.

The definition of the written and unwritten code is, however, not as clear as it might be made, judging by the means taken to promote individual gain by publicity.

The need of a true definition is nowhere more apparent than the difference made in the code of ethics between the sign on the door and the card in the daily newspaper. These may for all practical purposes be exactly alike, yet the one is accepted as very proper, if confined within certain limits as to size, while the other is usually regarded as very unprofessional. Exactly why a sign with Dr. John Doe, Dentist, placed to inform the thousands who perambulate the street should be considered right, while Dr. John Doe, Dentist, in the columns of a daily paper should subject Dr. Doe to censure under the code, is not clear. It seems but a question of numbers.

We are not disposed to quarrel with the mythical personage alluded to; indeed, he may be found in excellent company in many lands, and his bold assertion, that in so doing he is not violating professional usages, may have much to sustain it.

The question involves nice distinctions in ethical law. Yet it must be apparent to the most careless observer that the motive which influences John Doe to send his card to the newspaper is not strictly professional. The idea is clearly manifested in the action that he desires to make his name familiar as a household word, and to do that quickly rather than wait for the recognition of ability to give him an assured position in the community.

Whether professional men are right or wrong in their antagonism to advertisements does not concern us at present; the fact is quite clear that in many ways the professional advertiser is more than equal to overcoming any code, however strictly drawn.

Dentistry has received its lessons in this respect from the mother of the healing art, medicine, and has made its laws to correspond. The correctness of this needs no affirmation here, and yet it must be said that the devoted followers of medical science are not by any means indifferent to the pecuniary value of publicity.

If the question be considered solely from the stand-point of an advertisement, pure and simple, there would, probably, be no one to defend it; but there will be a difference of opinion when the question is presented, "Can a professional man give to a reporter an account of a 'remarkable case' and permit his name to go into the journal as the 'successful operator'?" Again, if articles are prepared for the instruction of the lay public, and unsigned, are they not indirectly an effort to call attention to the writer as one eminently qualified to perform more satisfactory work than his fellows? Have we reached the altruistic age when men do this unselfishly? It may be true, and we would not be thought to be impugning the motives of any who have performed this work; but the fact remains that any effort in this direction must prove a failure as far as the laity is concerned, and can only end in the interest of the writer of the articles by the silent, effectual, and universal knowledge that Mr. So-and-So is preparing a series of papers for the daily press.

The question whether such productions benefit those they are intended to educate has long been under discussion. There are certain basal facts in dentistry and medicine which should be promulgated and given as wide publicity as possible; but further than this the effort must prove a dismal failure. The proper place to begin the instruction in the care of the teeth is in the primary school, and it would not be amiss if this were occasionally a subject for selection in the Sunday-school. The experience of the writer in this leads to the opinion that seed sown here has never fallen on barren soil. Children eagerly absorb any simple facts of this character, and they are never lost.

Codes of ethics may not be equal to meeting all direct or indirect violations of law, and it is clear that some professional men approach the border-line of empiricism very closely in permitting their names to appear constantly in the daily journals. Newspaper men have their weak places, but no one will charge any member of that fraternity with the habit of doing something for nothing; and when certain names are paraded in their columns week after week, on the slightest pretext, it is not astonishing that the question is asked, "How is this accomplished?" Whether it be right or wrong for the public to insinuate motives, possibly not present in the individual, it is invariably done, and professional character suffers in consequence.

A pleasant way of evading the professional law, more frequently seen in the past than the present, was the giving a dinner to one of the medical fraternity after a two months' sojourn in Europe. Of course, the recipient and all connected with it had space in the columns of the dailies without serious cost to the parties concerned.

The long interviews, detailing, with singular minuteness medical treatment in various epidemic diseases, together with the name in full of the interviewed, are supposed to be published solely in the interest of the public.

We have no idea that the means detailed to give notoriety will come to an end. There must always exist in all professions a certain number whose activities will not permit the slow advances which reputation will eventually bring. Their idea is that notoriety is an essential factor in personal progress, and who can blame them if they use the only means in their possession to obtain recognition. It is, however, not professional, let the motive be what it may. The dentist or doctor is worthless who fails to possess the highest skill obtainable at the period of his labor. This skill or knowledge can only be made apparent and available by accumulated evidence, and this requires time and persistent work. The advertisement of the day will not bring reputation, neither will it bring an increased clientele upon which he can depend. True skill in any line of work is of slow and oftentimes painful growth; but once attained, it makes the possessor master of his position in a pecuniary and professional sense.

